# 2153 The plasma contact system and its role in common variable immunodeficiency (CVID): An explorative study

**DOI:** 10.1017/cts.2018.136

**Published:** 2018-11-21

**Authors:** Tukisa Smith, Manish Ponda, Jan Breslow, Charlotte Cunningham-Rundles

**Affiliations:** 1 Rockefeller University; 2 Division of Clinical Immunology, Icahn School of Medicine at Mount Sinai

## Abstract

OBJECTIVES/SPECIFIC AIMS: Assess the presence of contact activation at baseline in sera from common variable immunodeficiency (CVID) patients with and without inflammatory complications compared with healthy controls. METHODS/STUDY POPULATION: CVID patients were recruited in the outpatient setting and the measurement of cleaved plasma HK (cHK) levels was determined by Western blot analysis, under reducing conditions, with quantitation of total and cHK bands using an Odyssey imaging system (Licor). One-way ANOVA test for differences among the 3 studied groups will be applied. Biomarkers C3, C4, C1 inhibitor levels and hs-CRP were also measured. RESULTS/ANTICIPATED RESULTS: Participant enrollment continues and to date, 9 CVID patients were studied, 7 with and 2 without inflammatory complications. Repeated determinations of cleaved HK% (cHK%) revealed an average of 1.20% (range: 0.46%–2.66%) in CVID patients with inflammatory complications and those without complications averaged 1.07% (range: 0.79%–1.35%). Healthy controls had an average cHK of 1.15% (range: 0.60%–2.10%). DISCUSSION/SIGNIFICANCE OF IMPACT: Cleaved kininogen detected in the sera of CVID patients was found at similar levels compared with healthy controls (cHK<5%). Findings suggest that systemic activation of the contact system might be absent in CVID, however, future considerations include developing detection methods for local tissue activation.

